# Multispectral Fluorescence Imaging Technique for On-Line Inspection of Fecal Residues on Poultry Carcasses

**DOI:** 10.3390/s19163483

**Published:** 2019-08-09

**Authors:** Youngwook Seo, Hoonsoo Lee, Changyeun Mo, Moon S. Kim, Insuck Baek, Jayoung Lee, Byoung-Kwan Cho

**Affiliations:** 1 Rural Development Administration, National Institute of Agricultural Sciences, 310 Nonsaengmyeong-ro, Wansan-gu, Jeonju-si, Jeollabuk-do 54875, Korea; 2 Department of Biosystems Engineering, College of Agriculture, Life & Environment Science, Chungbuk National University, 1 Chungdae-ro, Seowon-gu, Cheongju, Chungbuk 28644, Korea; 3 Department of Biosystems Engineering, College of Agriculture and Life Sciences, Kangwon National University, Chuncheon 24341, Korea; 4 Environmental Microbial and Food Safety Laboratory, Agricultural Research Service, U.S. Department of Agriculture, Powder Mill Rd. Bldg. 303, BARC-East, Beltsville, MD 20705, USA; 5 Department of Biosystems Machinery Engineering, College of Agricultural and Life Science, Chungnam National University, 99 Daehak-ro, Yuseong-gu, Daejeon 34134, Korea

**Keywords:** food safety, poultry inspection, online measurement, multispectral fluorescence imaging

## Abstract

Rapid and reliable inspection of food is essential to ensure food safety, particularly in mass production and processing environments. Many studies have focused on spectral imaging for poultry inspection; however, no research has explored the use of multispectral fluorescence imaging (MFI) for on-line poultry inspection. In this study, the feasibility of MFI for on-line detection of fecal matter from the ceca, colon, duodenum, and small intestine of poultry carcasses was investigated for the first time. A multispectral line-scan fluorescence imaging system was integrated with a commercial poultry conveying system, and the images of chicken carcasses with fecal contaminants were scanned at processing line speeds of one, three, and five birds per second. To develop an optimal detection and classification algorithm to distinguish upper and lower feces-contaminated parts from skin, the principal component analysis (PCA) and partial least square discriminant analysis (PLS-DA) were first performed using the spectral data of the selected regions, and then applied in spatial domain to visualize the feces-contaminated area based on binary images. Our results demonstrated that for the spectral data analysis, both the PCA and PLS-DA can distinguish the high and low feces-contaminated area from normal skin; however, the PCA analysis based on selected band ratio images (F630 nm/F600 nm) exhibited better visualization and discrimination of feces-contaminated area, compared with the PLS-DA-based developed chemical images. A color image analysis using histogram equalization, sharpening, median filter, and threshold value (1) demonstrated 78% accuracy. Thus, the MFI system can be developed utilizing the two band ratios for on-line implementation for the effective detection of fecal contamination on chicken carcasses.

## 1. Introduction

It is necessary to supplant the current manual inspection of fecal contamination on the surface of chicken carcasses with autonomous fecal contamination inspection system because human inspection has limitations when it comes to detecting diluted fecal contaminations. The technologies predominantly employed for the detection and isolation of contaminants from agricultural products are machine vision and spectroscopy. Machine vision techniques based on image processing algorithms are used for classifying and sorting agricultural products via surface inspection [1,2,3,4,5,6]. These techniques are useful for detecting foreign matter on the surface of agricultural products based on visible wavelengths; however, the internal quality and the molecular analysis of such substances cannot be assessed based on visible wavelengths. In contrast, spectroscopic methods such as near-infrared spectroscopy (NIRS) provide rapid evaluation of the internal qualities of agricultural products based on the vibrational motions of organic molecules. Thus, spectroscopic techniques have been applied for the evaluation of the internal quality of agricultural products such as species discrimination [7,8,9,10], nutrient analysis [11,12,13], and internal defect detection [3,14,15,16,17,18,19]. 

The multispectral imaging technique possesses the characteristics of both machine vision and spectroscopic techniques. In other words, it can obtain the spectral and spatial information of a sample. Due to this unique property, it has been used in a range of applications to identify defects or biological contaminants in a wide range of agricultural products [20,21,22,23,24,25,26,27].

In particular, previous studies on the multi/hyperspectral imaging system have demonstrated the potential of reflectance imaging and fluorescence imaging techniques to detect contaminants on poultry carcasses [24,25,26,27]. Reflectance imaging exhibited detection accuracy of 92.4% for the bodily waste from the duodenum [24]. In comparison, fluorescence imaging achieved a detection accuracy of 100% using the band-ratio images of the fecal residues. Although the fluorescence imaging technique has been used to detect organic poultry residues (i.e., chicken fat, blood, and feces) on stainless steel plates, no attempt has been made to use it for fecal detection on chicken carcasses [25,26]. 

The main advantage of the fluorescence imaging technique is that its sensitivity to fluorescence-active compounds allows detection of even low concentrations of contaminants [25]. Secondly, spectral imaging is usually affected by specular reflection from the glossy surface of samples; thus, it sometimes misrepresents the sample representation. However, this effect is avoidable in fluorescence imaging. Hence, fluorescence imaging has been used for quality safety analysis of a range of products in the agro-food sector [28,29,30]. 

Due to this, the present research aims to develop a fecal contamination detection system for chicken carcasses using real-time multispectral fluorescence imaging (MFI). A multivariate analysis technique was implemented to detect and classify poultry fecal matters, and to differentiate fecal contamination from the skin of the carcasses moving at three different speeds of conveyor line. Furthermore, image processing using RGB image was employed to visualize fecal contaminants on chicken carcasses. Optimal wavebands were proposed for contaminant detection, and the performance of the band ratio images based on optimal wavebands. 

## 2. Materials and Methods 

### 2.1. Fecal Matters from Chicken Organs

**Figure 1:**
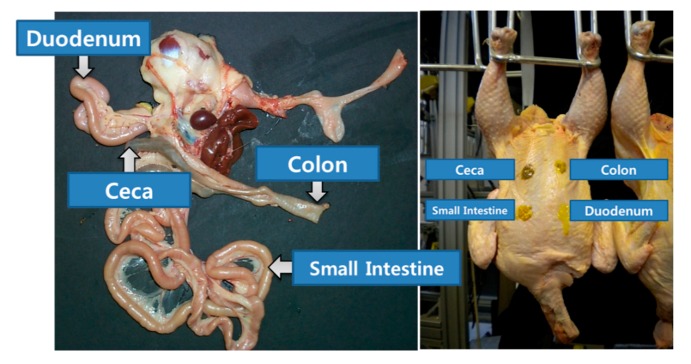
Body substances extracted from the chicken organs such as ceca, colon, small intestine, and duodenum.

The carcasses used in this study were of 30 chickens that were killed after being bred on soybean protein feed through standard practices for seven weeks. The carcasses and intestinal tracts were obtained from a poultry processing facility (Allen Foods Inc., Cordova, MD, USA). The chickens were deprived of food and water for 10 h before slaughter. Bodily waste, such as the fecal matters, was collected from their organs, specifically the ceca, colon, small intestine (SI), and duodenum (Figure 1). Four small spots were made on the skin of the chicken carcasses with the extracted substances, each spot comprising 50 μL drops. The mean dry matter contents of the ceca, SI, and duodenum, as measured by an oven-drying method, were 180 ± 21, 161 ± 21, and 149 ± 18 μg/g, respectively. Note that the dry matter content of the colon was not sufficient to be measured. In this study, intact feces were used to investigate the feasibility of the LED-induced fluorescence imaging technique as a practical tool for real-time poultry inspection. Color images were also acquired for the comparison using commercial digital camera (HCC-640NP, Honeywell Inc., Morris Plains, NJ, USA) and the light source system used was 100 W halogen lamps (Osram, Munich, Germany) directed at the stationary chicken carcass. 

### 2.2. Data Acquisition and Image Correction 

The multispectral fluorescence imaging (MFI) data were collected using a real-time imaging system for scanning fecal matters. The MFI system consisted of an electron multiplying charge-coupled device (Luca, Andor Technology, Concord, CT, USA), imaging spectrograph (VNIR, Headwall Photonics, Fitchburg, MA, USA) with a spectral wavelength range of 400–1000 nm, and two UV-A fluorescent lamps (ML-3500S, Mxindustrial Inc., Ivyland, PA, USA) with a central peak of 365 nm. A 400 nm long-pass filter (FEL0400, Thorlabs Inc., Newton, NJ, USA) was placed in front of objective lens (Rainbow S6X11, International Space Optics, S.A., Irvine, CA, USA) to prevent transmission of light lesser then approximately 400 nm and thus allowing only fluorescence signals to be collected while eliminating the reflectance light to reach the detector. The multispectral image was acquired with a line-scan method, also known as a push-broom method. The moving sample was scanned line-by-line with a multispectral information for each pixel along the length of every spatial line. The imaging data were displayed via a graphical user interface using Visual Basic (Ver. 6.0, Microsoft, Redmond, WA, USA). 

**Figure 2:**
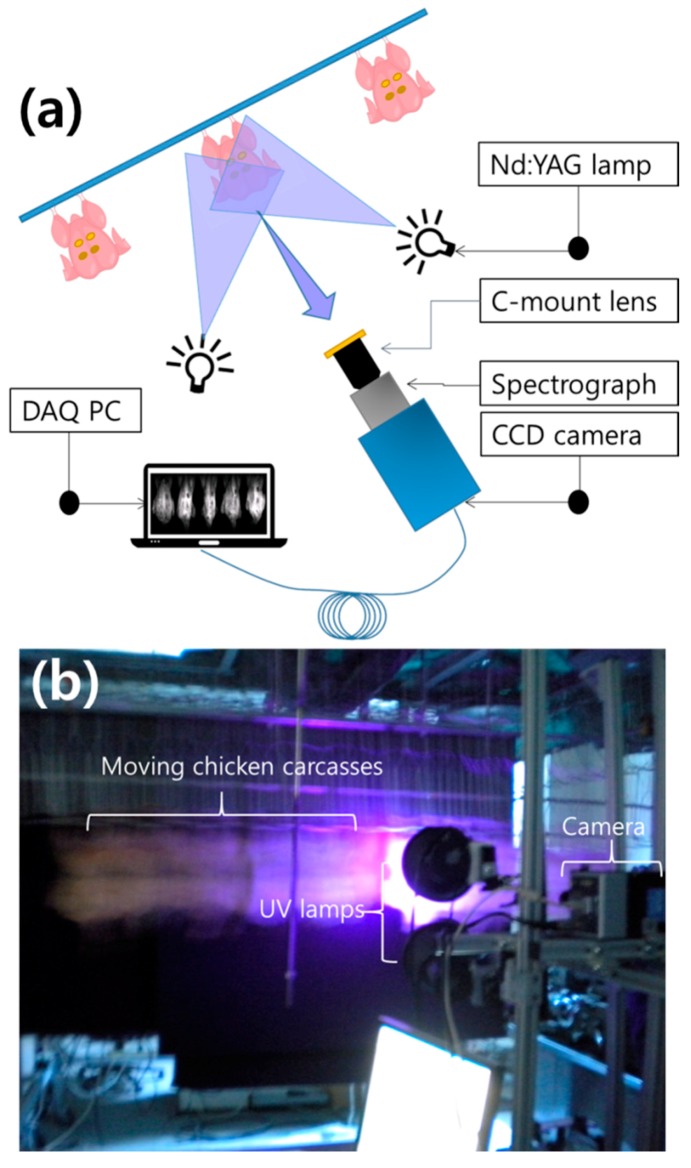
Schematic of the multispectral fluorescence imaging system (**a**) and a real-time multispectral fluorescence imaging system for detecting fecal matters on chicken surface (**b**).

Figure 2 shows the schematic and photo of the MFI with an inspection facility which was built in our laboratory that varies the speed of scan as 1 bird/s, 3 birds/s, and 5 birds/s. The fluorescence images were obtained by scanning each chicken with four fecal spots on its skin surface in the range 410–690 nm, with approximately 11 nm resolution between the contiguous bands, over a total of 27 bands. 

The images were measured in three speeds. Single online scanning with ten poultry images at three scanning speeds: 1, 3, 5 birds/s; the samples were tagged {sample#1, sample#3, sample#5}. Three replicas of each sample {sample#1A, sample#1B, sample#1C} were carried out. Each sample image, for example, sample#1A, comprised ten chickens; thus, a total of 30 chickens were used for the MFI imaging. Two sample images, out of three, were selected as the calibration data for data analysis, and the other sample images were used for the validation of the developed algorithms. 

### 2.3. Analysis of Fluorescence Spectra

To develop an efficient classification model for fecal matters on chicken carcass, we used sample compositions with four spots of fecal contaminations on the chicken carcasses. Two spots on the upper part were tagged the “upper-ROI”, the other two spots on the bottom were tagged the “bottom-ROI”; and the skin area between the two groups was tagged “skin-ROI”. Here, “ROI” refers to the region of interest within the rectangle (Figure 4 has five colored rectangles, each representing a ROI boundary). Fecal matters from four regions of the intestine were coated onto the skin; these were grouped into two ROIs: The upper-ROI, represented as a red square, contained fecal matter from the ceca and colon, while the bottom-ROI, the blue square, contained fecal matter from the small intestine and duodenum. The MFI spectra were extracted from three groups, and the selected number of pixels of each ROI ranged from 480–700 pixels. 

To reduce the data dimension and interpret the original dataset, the principal component analysis (PCA) was implemented [31,32,33]. The collected spectral data of all the three regions (upper and bottom-ROI, and skin-ROI) were saved in a matrix (X) to represent the data of the feces and skin from the chicken samples. The PCA, a representative and unsupervised linear dimensionality reduction algorithm, was applied to decompose the spectral data in matrix X into a loading matrix (L) and a score matrix (S). More specifically, X is assumed to be an N × K spectral data matrix, L is an N × A matrix of score vectors, and S is a K × A matrix of loading vectors; where N is the number of examined samples, K is the number of variables, and A is the number of principal components (PCs). 

The data analysis of the spectral data was carried out using a multivariate analysis method, the partial least square discriminant analysis (PLS-DA). The PLS-DA is based on the partial least squares regression algorithm; it assigns artificial numbers according to class, for instance, skin as zero, and fecal spots as one [33,34]. Using an efficient beta coefficient of PLS-DA which projects to unknown single spectra of ROI, we can obtain a digit, and classify the result as skin or fecal spot, according to the discriminant criteria (in this case, 0 as skin and 1 as fecal spots). 

To develop an optimal preprocessing method, three pre-processing algorithms, multiplicative scatter correction (MSC), and the first and second derivatives (D1 and D2) based on the Savitzky–Golay algorithm are utilized and compared to results of the classification accuracy [35]. The overall classification accuracy and Cohen’s kappa coefficient was used to describe the classification performance of each model [36]. Accuracy can be determined as the mean value of the sensitivity and specificity of each group, A and B, in a binary classification [0, 1], where sensitivity is defined as the ratio of correctly predicted samples to the sum of the samples of group A, denoted as 0. Specificity is the ratio of correctly predicted samples to the sum of the samples of group B, denoted as 1. The accuracy and kappa coefficient range from 0 to 1; closer to 1 means better accuracy. Spectral analysis and results visualization were conducted using R (ver. 3.3.2, Vienna, Austria), a software environment for statistical computing and graphics.

### 2.4. Analysis of Multispectral Fluorescence Image

**Figure 3:**
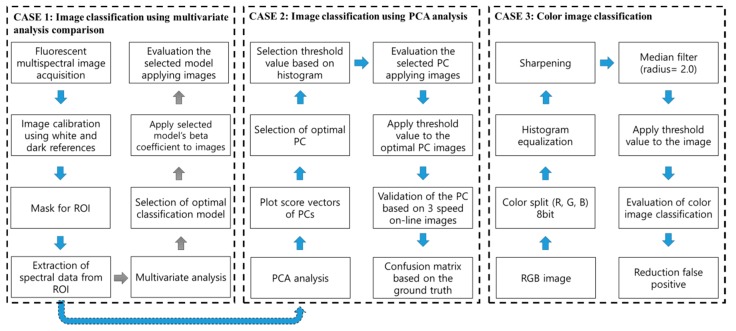
Schematic flowchart for multispectral fluorescence image acquisition and image classification. CASE1 shows data acquisition and spectral classification using multivariate analysis and CASE2 shows image classification based on the optimal principal component (PC). CASE3 is for color image classification.

The schematic flow chart of the image acquisition and classification analysis process showing the development of a classification algorithm based on spectral data is presented in Figure 3. CASE1 is the multispectral fluorescence image acquisition and spectral analysis process based on multivariate analysis methods. In the spectral analysis process, a mean plot, and score vectors according to PCA were used. 

CASE2 is the image classification algorithm based on the spectral analysis results that were implemented on the fluorescence images. A histogram of the calibration data, using PCA, was applied to segment the fecal spots from the background. The resultant PC images were enhanced with post image processing methods, such as histogram equalization, median filter, and sharpening. CASE3 is the image classification based on the RGB image. Image processing methods such as histogram equalization and median filter were implemented to detect and isolate fecal spots on the chicken surface. 

The threshold was calculated with multiple threshold algorithms based on ImageJ [37]. The simplest method for obtaining the threshold is constructing a histogram, which sums up all the pixel values in a picture, and selecting an optimal value among the cumulated histogram. A supervised manual method might achieve an optimal threshold for eliminating the background or distinguishing the target. 

Image correction and segmentation, spectral data extraction, and data analysis were performed using MATLAB (ver. 2011, The MathWorks, Inc., Natick, MA, USA). 

## 3. Results and Discussion

### 3.1. Spectral Characteristics

**Figure 4:**
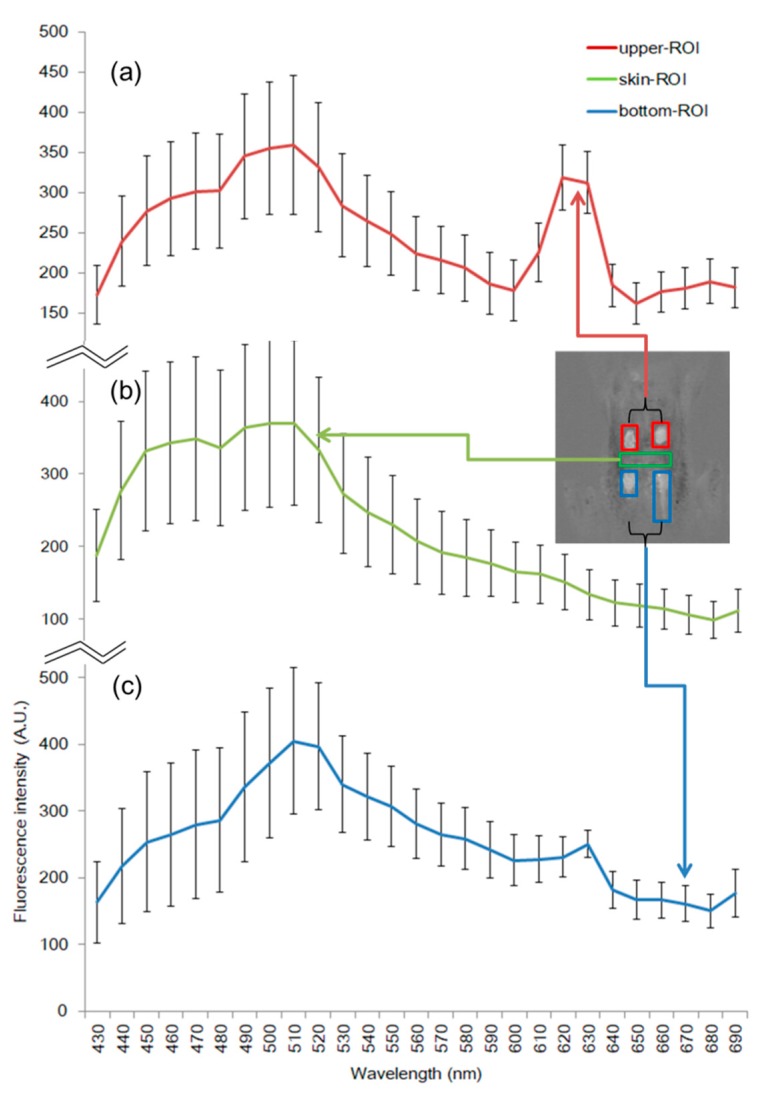
Mean and standard deviation plot of three ROI groups from upper/bottom-ROI (fecal spots) and skin-ROI. Upper-ROI (red line, **a**) and bottom-ROI (blue line, **b**) represent ceca, colon spots, and small intestine, duodenum spots. Skin-ROI (green line, **c**) covers up whole pixels of skin image.

Three ROI groups were selected, and their spectral data were analyzed to investigate the spectral characteristics of the fecal spots. Figure 4 visualizes a mean plot with 27 wavebands of the three spots with three colored lines, red, green dashed line, and blue dotted line, representing the upper-ROI, bottom-ROI, and skin, respectively, with wavelength ranging from 430 nm to 700 nm. 

In the range of 490 to 515 nm, the three groups show similar pattern such as signal valley at around 490 nm and slope ascension at approximately 515 nm; however, the fluorescence intensities vary. The upper-ROI peaks around 620 to 640 nm. Furthermore, the bottom-ROI has a comparatively small peak near 630 nm. In comparison, the skin-ROI exhibits no significant peak through 500 to 680 nm. The fluorescence values close to 630 nm can be a criterion threshold value for detecting fecal materials from chicken skin. This result shows that an emission peak at 635 nm, with excitation at 411 nm, can be observed from the various parts of the digestive tract, including the ceca, colon, duodenum, and small intestine, as reported in previous study [24]. The poultry fecal matter extracted from the digestive organs possibly contains blood substances, such that both the upper and bottom-ROI may reveal their fluorescence peaks around 630 nm. Although the spectral absorbance bands of pure myoglobin such as oxymyoglobin, deoxymyoglobin, and metmyoglobin are similar but apparent, it has high absorption optical density at ~555, ~578, and ~628 nm in the range of 500–700 nm, respectively [38]. It was reported that myoglobin is one of the iron ions; thus, it carries oxygen and gives meat color, using a protein of meat, protoporphyrin IX (PPIX) [39]. They demonstrated that the PPIX solutions extracted from chicken meat had fluorescence emission at 631 nm, with excitation at 405 nm. Another report indicates that strong emission peak is observed at around 635 nm, with excitation peak at 405 nm, which can be due to the PPIX in the chicken meat [40]. The result is similar to the excitation spectra measured at emission maxima in the previous study [24]. The excitation wavelength influencing on 635 nm peak spreads in between 360 and 430 nm as shown in the fluorescence emission–excitation matrices in the other study [41]. The results indicate that UV-A light with the center wavelength of 365 nm used in this study can produce an emission peak of the fecal matters at around 635 nm even though the intensity is relatively lower than that with 410 nm excitation. Thus, it is a reasonable assumption that the upper-ROI and bottom-ROI have the features of fluorescence of PPIX. 

### 3.2. Spectral Data Analysis 

#### 3.2.1. Spectral Analysis Using PCA

**Figure 5:**
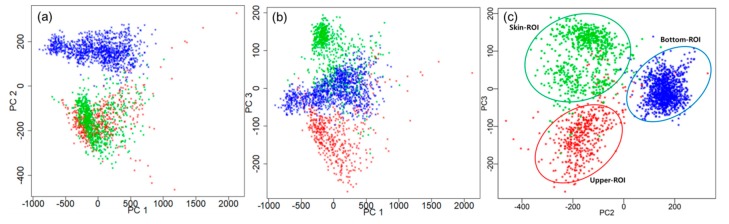
Score plot of three groups with PC2 and PC3 space in 2 dimensional. Red asterisks denote upper-ROI, blue ones are bottom-ROI, and green ones are skin-ROI.

PCA was carried out to find the optimal threshold for the detection and isolation of the fecal spots from the skin fluorescence spectra. Figure 5a–c illustrates the score vectors of the PCA in 2D space using the spectral data. PC2 and PC3 illustrate three groups that are distinguishable from each other (Figure 5c). In the score plot, the red asterisk signifies the samples of spectral data from the upper-ROI, the blue ones signify the bottom-ROI, and the green ones signify the skin-ROI. The spectral domain shows that the three groups are highly separable. 

#### 3.2.2. Spectral Classification Using PLS-DA

Table 1 shows the multivariate analysis results based on spectral data using PLS-DA, in accordance with preprocessing methods. To eliminate environmental noise, and find the optimal preprocessing method, three preprocessing methods and a method without preprocessing, noted as X, are compared. Without preprocessing, the PLS-DA has an accuracy of 97.6% and a kappa coefficient of 0.96 (Table 1). The first and second derivatives (D1 and D2) demonstrate similar accuracy results, 92.3% and 92.8%, respectively. The MSC exhibits reasonable separation result; in particular, skin-ROI is isolated from fecal matters. Others show distinguishable skin-ROI detection results with more than 96% accuracy. The mean spectrum exhibited a characteristic spectrum curve near 635 nm for both fecal matters and skin-ROIs (Figure 4). The spectral characters may be obtained from the classification results because the PLS-DA can handle multiple dependent categorical variables based on the maximization of the covariance between the independent variables (spectral data of three ROIs) and the dependent variables (i.e., classes) [42]. Overfitting or producing an over-optimistic model is common issue in PLS-DA. Usually, cross-validation is used to avoid overfitting and preprocessing for reducing noise from dataset. In this study, leave one out (LOO) cross-validation is used to avoid overfitting. Furthermore, we applied three preprocessing methods; however, the X method (non-preprocessing method) demonstrated the best accuracy. MSC uses the mean centered spectrum as an artificial baseline for scaling, thereby reducing scattering effects [43]. The first and second derivative methods have potential effects for reducing baseline influence according to derivative gap [44]. However, the preprocessing method does not always improve the classification performance in the case of a consistent dataset not influenced by the environment or hardware changes during measurement. 

### 3.3. Image Data Analysis 

#### 3.3.1. Color Image Classification 

**Figure 6:**
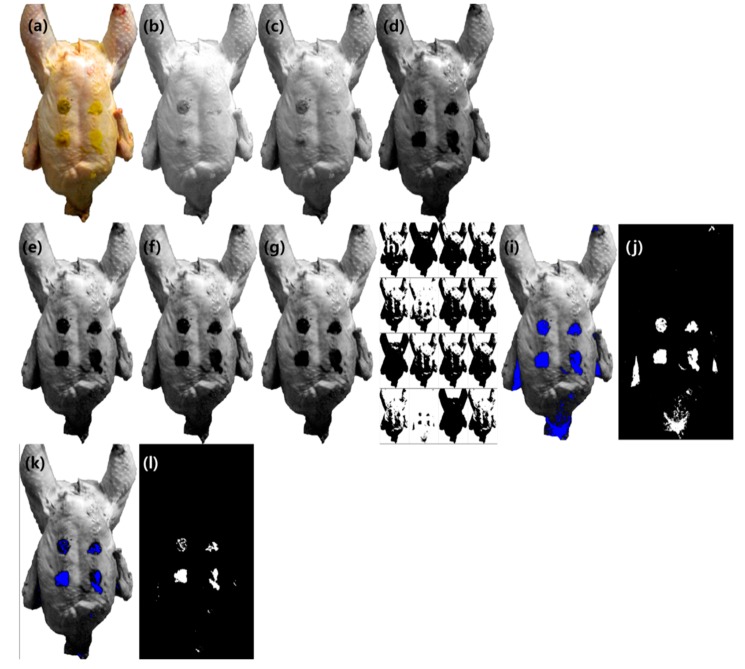
Result of color image classification. A RGB color image (**a**) was split into 8-bit RGB component as red (**b**), green (**c**), and blue (**d**) image. Blue image was selected as a reference image to find the optimal threshold. Image processing methods were applied such as histogram equalization (**e**), sharpening (**f**), and median filter (**g**) (radius = 2.0). The result of auto threshold method and Shanbhag algorithm (threshold = 17) were the most acceptable result (**h**–**j**). The revised threshold value (threshold = 1) was employed and its pseudo colored and black/white result image (**k**,**l**).

Image processing methods were used to detect and isolate fecal spots from a chicken carcass. To obtain the optimal threshold value, the blue image was selected among the RGB components, and several image processing methods, such as histogram equalization (saturated pixels: 0.3%) and sharpening and median filter (radius = 2.0), were employed. Figure 6 shows the classification results according to the threshold value. Pertaining to the auto threshold method [45], of the 16 algorithms, the Shanbhag algorithm [46] yielded reasonable result (threshold value = 17), and its pseudo colored (Figure 6i) and black/white image (Figure 6j) were demonstrated. As shown in Figure 6i–j), four spots were detected with 100% accuracy; however, redundant false positive pixels were a challenge. To reduce false positive pixels, we revised the threshold value from 17 to 1 and employed the revised threshold value to the image (Figure 6k,l). Using the developed algorithm and the threshold value, an accuracy of 78% was achieved with 29 samples (data not shown). Thus, the revised threshold value (1) was optimal for reducing false positive pixels from chicken images.

#### 3.3.2. Image Classification Using Band Ratio

**Figure 7:**
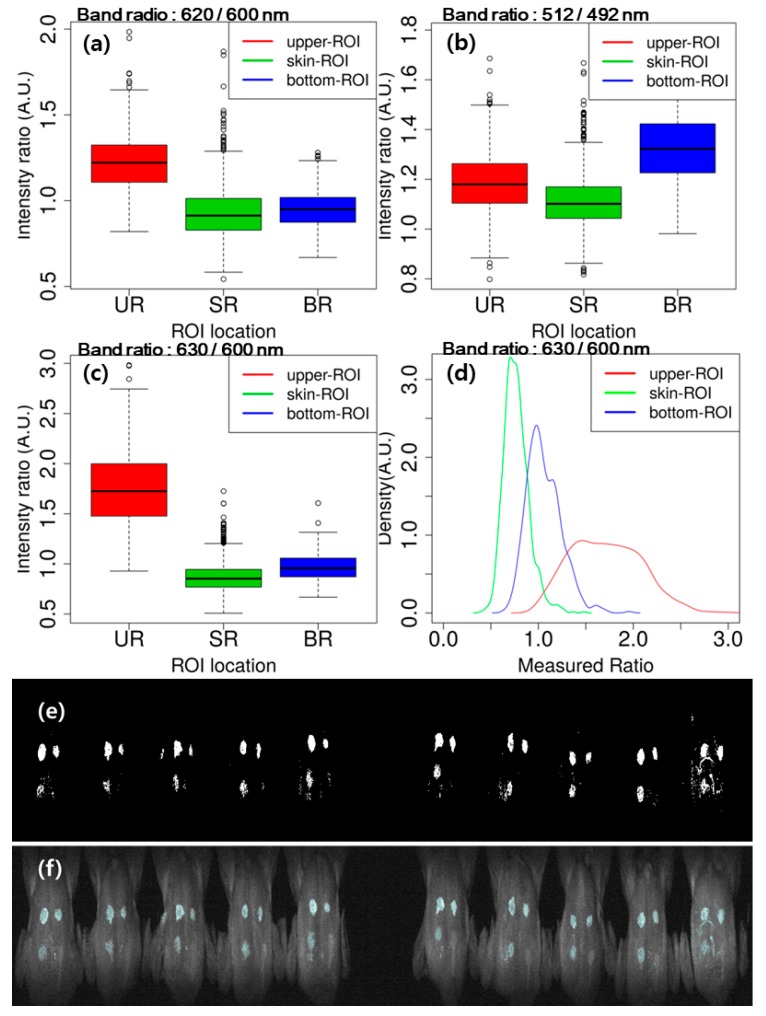
Band ratios for discrimination of upper-ROI (UR), skin-ROI (SR), and bottom-ROI (BR) with the fluorescence intensity of 620 and 600 nm (**a**), 512 and 492 nm (**b**), and 630 and 600 nm (**c**). Density plot (**d**) of the kernel density of band ratio 630/600 (**c**). Black and white image of the band ratio 630/600 nm (**e**) and its projection onto the sample images (**f**).

Figure 7 illustrates the calculation result of the band ratio between two wavelengths for group classification. Figure 7a,b shows the results of band ratio such as 620/600 nm and 512/492 nm for the classification of the upper-ROI and bottom-ROI, respectively, and Figure 7c illustrates the ratio between 630 and 600 nm for the three groups. The band ratio of 630/600 nm shows the potential for fecal-spots identification. The density plot (Figure 7d) visualizes the histogram of each group’s data for more detailed explanation on the band ratio of 630/600 (Figure 7c). In Figure 7d, the red line represents the histogram of the upper-ROI; the blue line is the bottom-ROI, and the green line is the skin-ROI. The red and green lines are clearly distinct from each other, whereas, the blue line lies between two lines, making it indistinguishable from the former groups. In other words, although ratio 630 and 600 nm can easily isolate the upper-ROI and skin-ROI, identification of the bottom-ROI (blue line) might be affected by the other groups. Figure 7e shows the binary black and white threshold image of band ratio 630/600 nm, and its projection onto the sample images is shown in Figure 7f. The upper-ROI spots are distinctive from skin, while the bottom-ROI is not as clearly distinct from skin; thus, more efficient and improved image classification methods are necessary. 

#### 3.3.3. Image Classification Using PLS-DA

**Figure 8:**
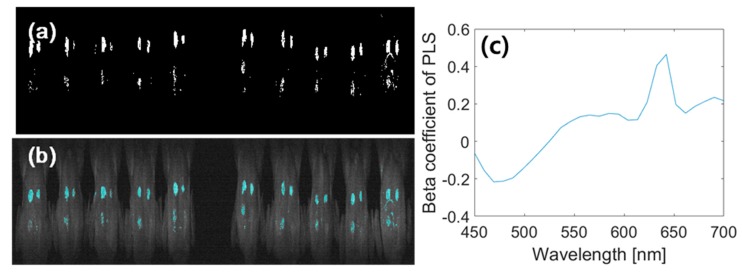
The resultant binary image of partial least square discriminant analysis (PLS-DA) to the multispectral images (**a**) and its projection to the sample image with a pseudo color (**b**). Beta coefficient of PLS-DA of 2nd latent variable (**c**).

Based on the results of the PLS-DA in spectral analysis, the beta coefficient of the PLS-DA is applied to the entire spectra of the integrated multispectral fluorescence image and its binary black and white image (Figure 8a,c) is obtained. Figure 8b shows the pseudo colored image projected onto the integrated fluorescence image. Four spots are detected with 75% accuracy, whereas the image result shows redundant pixels around the four spots. The PLS-DA demonstrated remarkably good accuracy that may provide a good interpretation of the relationship between the two fecal spot groups and skin. However, the projected images showed some errors in the bottom-ROI because latent variables may not represent the linear combinations between the original wavelengths (MFI data) and response variables (three groups) [47]. In addition, an advantage of developing the prediction model using the PLS-DA with a low number of variables is its prediction accuracy, compared to other approaches [41]. The PLS-DA demonstrated more accurate detection of fecal spots on the chicken carcass surface (accuracy: 92.5%) compared to the band ratio method (accuracy 75%), as illustrated in Figure 7f.

#### 3.3.4. Image Classification Using PCA

**Figure 9:**
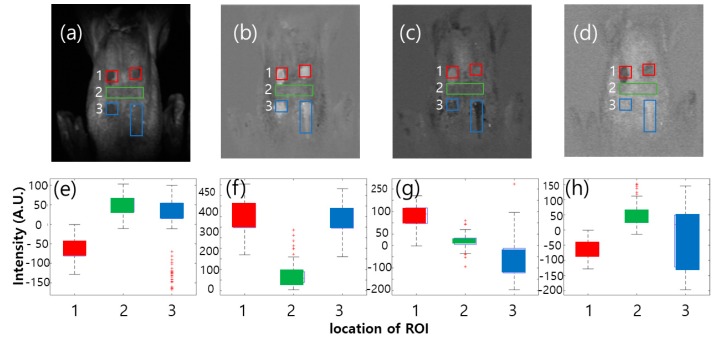
PC images of on-line scan multispectral fluorescence imaging (MFI) images for chicken with fecal spots. PC images (**a**–**d**) and four boxplots (**e**–**h**) collected pixels from red (1), green (2), and blue (3) rectangles.

According to Figure 5, the PCA assumed that a potential multivariate analysis method was used for the classification of the three groups. The PCA is a well-known data-dimension reduction technique; the benefits of PCA to image processing include reduction of processing time and memory maximization. PCA is applied to the multispectral fluorescent images. Figure 9 shows four PC images (a–d) and its boxplot (e–h). Among them, the PC2 image (Figure 9b) shows four spots clearly distinguishable from the chicken skin surface. The PC4 image (Figure 9d) presents the upper-ROI clearly; however, the bottom-ROI is not as distinct. To verify the human analysis results, simple statistical measures, such as mean and standard deviation, were deployed to compare the spectral intensities of the four PC images. Figure 8e–h shows the results of the comparison of the group pixels, with the red, green, and blue rectangles illustrating the group area. To select the optimal PC image for fecal spot isolation from the four PCs, spectral information was extracted from the four fecal spots red rectangle (Figure 9a) as upper-ROI, blue rectangle (Figure 9a) as bottom-ROI, and green rectangle (Figure 9a) as skin-ROI, and used to calculate the mean and standard deviation, as shown in the boxplot in Figure 9e–h. 

**Figure 10:**
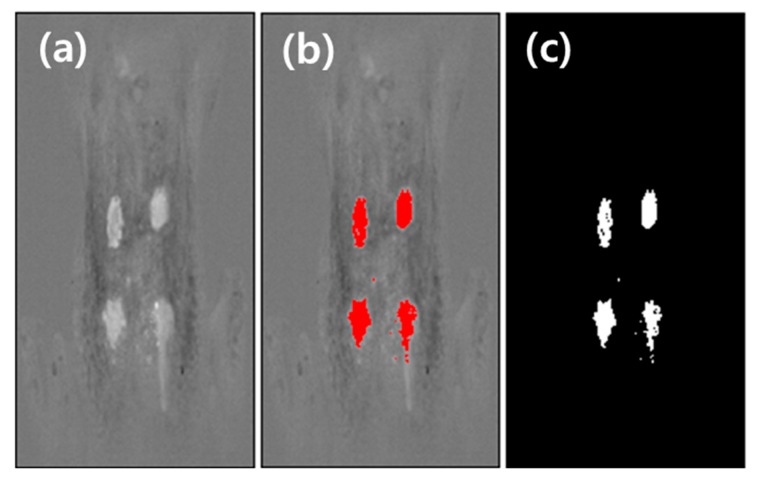
Threshold algorithms with PC2 image using ImageJ. PC2 image (**a**) was applied to determine the threshold value (155) using the Huang method. The threshold employed to the PC2 image (**b**) and its binary image (**c**).

Using the PC2 image, the threshold value (155) was set based on the Huang’s fuzzy thresholding method according to auto threshold technique [48]. Figure 10b,c shows the threshold value applied to the image and its binary image, respectively. 

**Figure 11:**
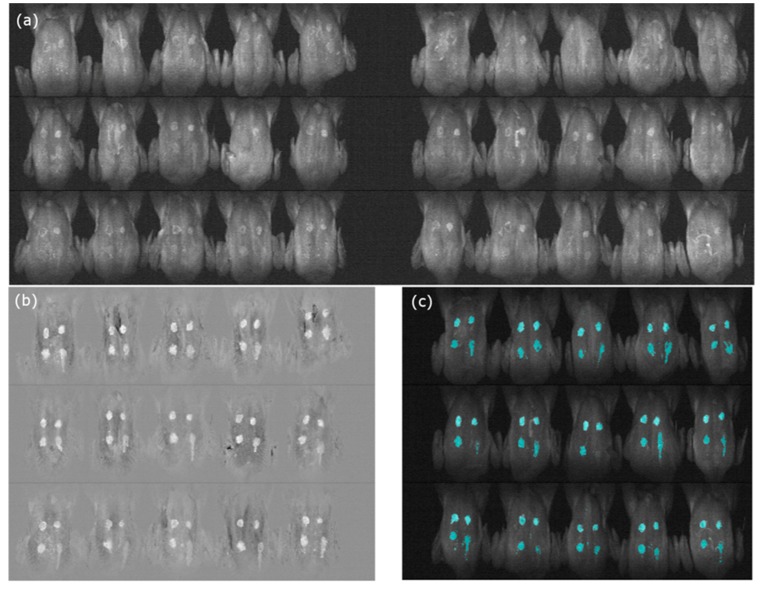
In order to find the optimal threshold value 30 samples (**a**) were used. PC2 employed (**b**) and selected the optimal threshold value (155) and its validation using the threshold value with artificial color (**c**).

Evaluation was carried out using a sample mosaic picture that consisted of three sample plates (Figure 11a); half of the data was used for the calibration of the classification methods (Figure 11b) and the other half for validation (Figure 11c). The PC2 image shows the representative characteristics of the fecal spots, demonstrating that the PCA has potential for fecal spot detection and image classification. Furthermore, the PCA is a representative dimension-reduction method and grouping method that does not require a prior knowledge of samples in the original dataset [40]. Therefore, only the few variables that can describe the correlation between samples are deployed in the classification.

**Figure 12:**
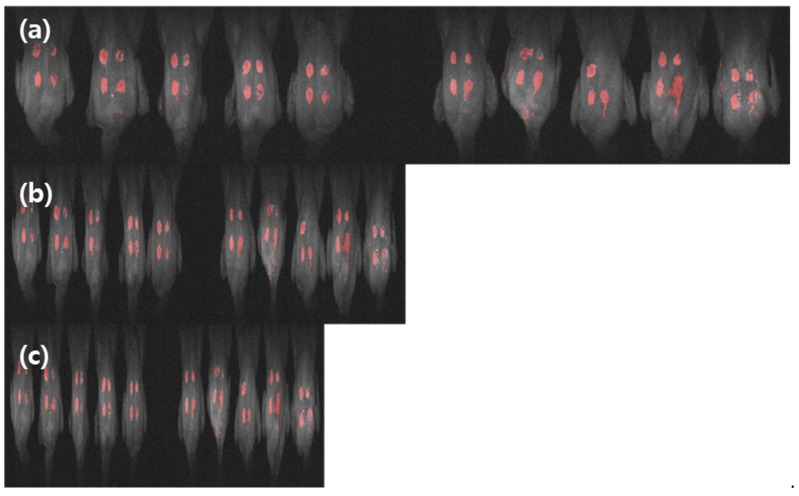
Evaluation of principal component analysis (PCA) for fecal contamination detection on chicken carcasses moving at three different speeds of conveyor line: (**a**) 1 bird/s, (**b**) 3 birds/s, and (**c**) 5 birds/s. PCA detection and isolation accuracy for fecal spots on chicken carcasses is 97.5%.

Figure 12 shows the PCA-implemented images of the online multispectral fluorescence images of the three replicas obtained at the rate of 1 bird/s, 3 birds/s, and 5 birds/s. With the same threshold, fecal spot detection was successfully performed on the three online samples, and 39 out of 40 fecal spots were isolated (accuracy: 97.5%) using the PC2 criteria. This indicates that fecal spots can be detected and isolated from poultry skin on a real-time conveying system using multispectral fluorescence imaging. PCA results may vary depends on the measurement environment because it is an unsupervised classification method. Hence, the sorting machine used in the processing facility should be always calibrated before using, which is the thumb of rule in the real field application. The classification model shown in this study may need a fine tuning to be used in a difference environment.

## 4. Conclusions

In this study, a fecal contaminant detection technique for poultry carcasses using online multispectral fluorescence images based on multivariate analysis technique and image processing algorithms was investigated. Four small spots were made on the chicken skin using poultry bodily wastes extracted from the digestive system, specifically, the ceca, colon, duodenum, and small intestine. 

Our results indicate that multispectral fluorescence imaging (MFI) has good potential for the detection of feces on poultry carcasses, and it could be an alternative to the current manual inspection method in automated poultry processing plants. Further, PCA is effective for isolating fecal matter from the chicken carcass, as it decreases the calculation time by reducing the dimensions of the data. Color image processing also exhibited a potential for detecting and isolating four residuals from stationary chicken carcasses. However, the revised threshold value (17 → 1) revealed a trade-off between the enhancement of detection accuracy (false positive reduction) and the reduction of the selected pixels of the upper-ROI (less accuracy). To evaluate the performance of the developed fecal contaminant detection algorithm with regards to chicken carcasses on motion, further study is necessary. Furthermore, to develop a robust fluorescence based inspection system for the detection of various types and levels of diluted fecal contaminants, additional research with poultry carcasses fed on different feedstuff is necessary.

## Figures and Tables

**Table 1:** Classification result using multivariate analysis method according to pre-processing methods.

	Pre-Processing	Bottom-ROI	Upper-ROI	Skin-ROI	Accuracy	Kappa
**PLS-DA**	X*	92.4%	97.8%	99.8%	97.6%	0.96
	MSC	81.0%	86.7%	99.7%	90.1%	0.84
	D1	80.5%	96.8%	96.0%	92.3%	0.88
	D2	79.7%	98.2%	96.3%	92.8%	0.89

X*: Without pre-processing, MSC: multiplicative signal correction, D1: 1st derivative, D2: 2nd derivative.
